# Evaluation of the Prognostic Value of Impaired Renal Function on Clinical Progression in a Large Cohort of HIV-Infected People Seen for Care in Italy

**DOI:** 10.1371/journal.pone.0124252

**Published:** 2015-05-01

**Authors:** Alessandra Bandera, Andrea Gori, Francesca Sabbatini, Giordano Madeddu, Stefano Bonora, Raffaella Libertone, Claudio Mastroianni, Paolo Bonfanti, Antonella d'Arminio Monforte, Alessandro Cozzi-Lepri, Icona Foundation Study Group

**Affiliations:** 1 Division of Infectious Diseases, Department of Internal Medicine, San Gerardo Hospital, University of Milano-Bicocca, Monza, Italy; 2 Department of Infectious Diseases, University of Sassari, Sassari, Italy; 3 Department of Infectious Diseases of the University of Torino, Amedeo di Savoia Hospital, Torino, Italy; 4 National Institute for Infectious Diseases Lazzaro Spallanzani, Rome, Italy; 5 Department of Infectious and Tropical Diseases, Sapienza University, Polo Pontino, Latina, Italy; 6 Infectious Diseases Unit, Alessandro Manzoni Hospital, Lecco, Italy; 7 Clinic of Infectious Diseases and Tropical Medicine, University of Milan, San Paolo Hospital, Milan, Italy; 8 University College London, London, United Kingdom; University of São Paulo School of Medicine, BRAZIL

## Abstract

Whilst renal dysfunction, especially mild impairment (60<eGFR<90 ml/min), has been often described in HIV-infected population, its potential contribution to HIV evolution and risk of cerebro-cardiovascular disease (CCVD) has not been clarified. Data from HIV-1 infected patients enrolled in the Italian Cohort of Antiretroviral-Naïve (Icona) Foundation Study collected between January 2000 and February 2014 with at least two creatinine values available. eGFR (CKD-epi) and renal dysfunction defined using a priori cut-offs of 60 (severely impaired) and 90 ml/min/1.73m^2^ (mildly impaired). Characteristics of patients were described after stratification in these groups and compared using chi-square test (categorical variables) or Kruskal Wallis test comparing median values. Follow-up accrued from baseline up to the date of the CCVD or AIDS related events or death or last available visit. Kaplan Meier curves were used to estimate the cumulative probability of occurrence of the events over time. Adjusted analysis was performed using a proportional hazards Cox regression model. We included 7,385 patients, observed for a median follow-up of 43 months (inter-quartile range [IQR]: 21-93 months). Over this time, 130 cerebro-cardiovascular events (including 11 deaths due to CCVD) and 311 AIDS-related events (including 45 deaths) were observed. The rate of CCVD events among patients with eGFR >90, 60-89, <60 ml/min, was 2.91 (95% CI 2.30-3.67), 4.63 (95% CI 3.51-6.11) and 11.9 (95% CI 6.19-22.85) per 1,000 PYFU respectively, with an unadjusted hazard ratio (HR) of 4.14 (95%CI 2.07-8.29) for patients with eGFR <60 ml/min and 1.58 (95%CI 1.10-2.27) for eGFR 60-89 compared to those with eGFR ≥90. Of note, these estimates are adjusted for traditional cardio-vascular risk factors (e.g. smoking, diabetes, hypertension, dyslipidemia). Incidence of AIDS-related events was 9.51 (95%CI 8.35-10.83), 6.04 (95%CI 4.74-7.71) and 25.0 (95%CI 15.96-39.22) per 1,000 PYFU, among patients with eGFR >90, 60-89, <60 ml/min, respectively, with an unadjusted HR of 2.49 (95%CI 1.56-3.97) for patients with eGFR <60 ml/min and 0.68 (95%CI 0.52-0.90) for eGFR 60-89. The risk of AIDS events was significantly lower in mild renal dysfunction group even after adjustment for HIV-related characteristics. Our data confirm that impaired renal function is an important risk marker for CCVD events in the HIV-population; importantly, even those with mild renal impairment (90<eGFR<60) seem to be at increased risk of cerebro-cardiovascular morbidity and mortality.

## Introduction

HIV infected individuals, because of the availability of effective and well tolerated antiretroviral treatments (ART), show considerably prolonged survival and are now facing a new set of health challenges. Several studies have addressed non-HIV related diseases in HIV-positive population, identifying aging, antiretroviral treatments and HIV itself as leading causes of premature atherosclerosis vascular disease leading to increased cardiovascular disease mortality in these patients [1]. Accumulated evidence in the general population suggests that reduced kidney function exposes patients to an increased rate of cerebro-cardiovascular disease (CCVD) both in populations at high risk for CCVD and even in the absence of traditional risk factors [2–3].

Proportion of patients with HIV infection and renal impairment of any grade are variable in published studies, ranging between 5% and up to 32% in some cases and mostly represented by mild renal dysfunction (90<eGFR<60 ml/min) [4–7]. Mild renal dysfunction is also frequent in HIV-infected population seen for care in Italy with a prevalence of 25% in ART-naïve patients [8].

Previous analysis that investigated the association between kidney disease and the risk of CCVD in HIV-population showed that both an eGFR less than 60 ml/min and albuminuria were associated with higher risk of CCVD and heart failure [9]. Moreover, severe renal dysfunction has been associated with increased risk of HIV disease progression and even death in several studies [10–13]. However, most of these studies evaluated patients not receiving ART or they have been conducted in the early highly active antiretroviral therapy era [9], thus concerning settings with a greater incidence of AIDS events. These studies often involve populations of patients living in settings where antiretroviral treatments are not largely available or there is limited access to healthcare [11–12], such as sub-Saharian Africa, exposing people to a greater risk of HIV progression. In addition, none of these studies focused on mild renal dysfunction and its predictive role on clinical outcome has not been assessed.

We, therefore, aimed to use the data of patients enrolled in the Icona Foundation Study to evaluate the prognostic value of reduced eGFR (both <60 ml/min and 60–90 ml/min) in predicting the occurrence of CCVD, AIDS defining diseases and consequent deaths over prospective follow-up.

## Methods

### Patient population

This analysis includes data from HIV-1 infected patients enrolled in the Italian Cohort of Antiretroviral-Naïve Patients (Icona) Foundation Study and collected after January 2000 (date of routine collection of creatinine for all patients). All information recorded in the database up to February 1st 2014. According to protocol inclusion criteria, patients had to be > 18 years old and start ART when they were naïve to antiretrovirals. In addition, for inclusion in this analysis, they had to have at least two creatinine values available after January 2000.

The ICONA Foundation Study is an Italian multi-centric prospective observational cohort study of HIV-1-positive persons enrolled since 1997. CD4+ T cell count, HIV-1-RNA, HCV antibody, HBV surface antigen and antibody and creatinine are systematically recorded every 6 months. Creatinine is measured using a method traceable to IDMS (upper limit of normal 1.3 mg/dL) at the local laboratories of the various sites. Clinical and laboratory data and data regarding any drug taken by the patient, either antiretroviral or else, are collected for all participants and recorded using an electronic data collection form (www.iconafoundation.it). All data are updated at the occurrence of any clinical event and, in the absence of such an event, at least every 6 months. AIDS-defining diseases and CCVD events captured by medical record abstraction are recorded in the database at the date that this diagnosis is confirmed.

The eGFR was used to identify patients in the cohort with potential renal dysfunction. To better estimate renal function, eGFR was calculated using the CKD-epi formula: GFR = 141 X min(Scr/κ,1)^α^ X max(Scr/κ,1)^-1.209^ X 0.993^Age^ X 1.018 [if female] X 1.159 [if black]. Where Scr is serum creatinine (mg/dL), κ is 0.7 for females and 0.9 for males, α is −0.329 for females and −0.411 for males, min indicates the minimum of Scr/κ or 1, and max indicates the maximum of Scr/κ or 1.

This method of calculation recently emerged as a useful filtration marker even in HIV positive population, because of reducing bias for all traditional cardiovascular risk factors [14–16].

All individuals signed an informed consent prior to enrollment and the study was approved by the Ethics Committee of each participating institution that are listed in the Acknowledgments.

### Outcome

The outcomes of interest were the occurrence of newly diagnosed cerebro-cardiovascular events or AIDS-related events. Only the first of these diagnoses were counted as events and relapses have been ignored in the analysis. The cerebro-cardiovascular endpoint included the following events: angioplasty, coronary artery bypass, cardiac arrhythmia, cerebral haemorrhage, carotid endarterectomy, cerebral stroke, dilated cardiomyopathy, other cardiac surgical procedures, death due to cardiovascular events. HIV-related events were defined as the occurrence of a new AIDS-related opportunistic infections or neoplasms (as defined by the Center for Disease Control and Prevention 1993 classification) and occurrence of AIDS-related deaths.

### Statistical analysis

Baseline was defined as the date of the first of the two consecutive creatinine values measured after January 2000. Patients were grouped according to eGFR values calculated at baseline using the a priori defined cut-offs of 60 (severely impaired, if at least one value < 60 ml/min) and 90 ml/min/1.73m^2^ (mildly impaired, if at least two values between 60–89 ml/min). Characteristics of patients were described after stratification in these groups and compared using chi-square test (categorical variables) or Kruskal-Wallis test comparing median values (quantitative measures).

Follow-up accrued from baseline up to the date of the event of interests or last available clinical follow-up visit. Rates of events have been calculated as number of new diagnoses occurring after baseline divided by number of person years of follow-up (PYFU). Confidence intervals for these estimates were calculated using a Poisson approximation.

Survival analysis has also been employed. Kaplan Meier curves were used to estimate the cumulative probability of occurrence of the events by longer time from baseline. Log-rank test was used to compare survival of patients according to different eGFR strata. A multivariable Cox regression analysis was performed to control for confounding. A sequential manual adjustment was used so that estimates were first adjusted for demographic factors (i.e. age, gender, mode of HIV transmission, nationality and calendar year at baseline). Then they were further adjusted for other set of covariates such as traditional risk factors for CCVD disease and other clinical parameters (i.e. smoking, diabetes, use of blood pressure and lipids lowering drugs, total cholesterol, blood glucose and high density lipoprotein cholesterol [HDL]). Finally a further adjustment was made by adding virus- host- and treatment—related factors such as use of ART, baseline CD4, CD8 count and nadir CD4 count, HIV RNA and co-infection with HBC/HCV into the model. Unless noted, only baseline values of time-varying covariates were included in the model. Relative hazards of cardiovascular disease according to defined eGFR strata have been estimated and tabulated together with 95% confidence intervals. eGFR was also examined in the log10 scale because the distribution was skewed in the raw scale. All statistics were 2-sided and a p-value ≤0.05 was considered as significant. SAS version 9.3 was used for all analyses.

## Results

### Study population

Seven thousand three hundred eighty-five patients were included in the study and observed over a median follow-up of 43 months (inter-quartile range [IQR]: 21–93 months).

According to baseline eGRF, patients were stratified in 3 different groups: 5,243 patients (70.9%) had normal renal function (eGFR>90 ml/min), 1,936 patients (26.2%) had mild renal impairment (eGFR between 60–89 ml/min in at least two consecutive measures) and 206 patients (2.7%) showed reduced renal function (eGRF <60 ml/min) at this single baseline measurement.

Median age was 36 years (IQR 31–42), 26.6% of patients were females, HIV transmission was mainly by heterosexual intercourses (40.1%). Non-Caucasian patients were poorly represented in our cohort (4.4%). Only a few patients had an AIDS diagnosis prior to the enrollment in the study (12.3%), consistently with the nadir of CD4+ T cells count (342/μl, IQR: 183–508). Comparing the 3 categories of patients according to baseline eGFR, a previous AIDS diagnosis was more frequent in patients with reduced renal function (29.1% and 12.9% among those with eGFR<60 ml/min and ≥90 ml/min, respectively, p<0.001). Despite the small representation of patients with a previous diagnosis of cerebro-cardiovascular disease (8.9%), the proportion was higher in participants showing greater reduction in renal function (27.2%, 11.5% and 7.2% among those with eGFR<60, 60–89, ≥90 ml/min, respectively, p<0.001). Patients with reduced renal function showed a lower median CD4 T cells count (361 [124–551], 453 [270–660], 449[288–646] cells/μl among those with eGFR<60, 60–89, ≥90 ml/min, respectively, p<0.001), and a lower nadir CD4 T cells count (200 [57–400], 340 [175–502], 348 [192–514] cells/μl in the eGFR<60, 60–89 and ≥90 ml/min groups, respectively, p<0.001). People with lower eGFR were also less likely to be ART-naïve (28.6%, 26.9%, 24.8% in eGFR<60, 60–89, >90 ml/min groups, respectively, p<0.001). Of note, HIV-infected patients with the worse baseline eGFR values also showed a higher prevalence of traditional cerebro-cardiovascular risk factors, such as diabetes (7.8% and 1.6% among those with eGFR<60 and ≥90 ml/min, respectively, p<0.001), hypertension (12.1% versus 2.0% among those with eGFR<60 and ≥90 ml/min, respectively, p<0.001) and dyslipidemia (total cholesterol median values 179 [145–215] and 167 [141–196] mg/dl among patients with eGFR <60 and ≥90 ml/min, respectively, p<0.001; HDL cholesterol median level 37 [29–49] and 42 [34–52] mg/dl in patients with eGFR <60 and ≥90 ml/min, respectively, p = 0.002). All characteristics of the patients stratified by baseline eGFR are reported in Table 1.

**Table 1:** Characteristics of patients by baseline eGFR.

		eGFR (CKD_Epi formula, ml/min/1.73m^2^)				
Characteristics		>90	89–60	<60	Total	p-value
		N = 5243	N = 1936	N = 206	N = 7385	
***Gender*, *n(%)***	<.001					
Female		1327 (25.3%)	576 (29.8%)	58 (28.2%)	1961 (26.6%)	
***Mode of HIV Transmission***, ***n(%)***	<.001					
IDU		1263 (24.1%)	360 (18.6%)	34 (16.5%)	1657 (22.5%)	
Homosexual contacts		1651 (31.5%)	593 (30.7%)	52 (25.2%)	2296 (31.1%)	
Heterosexual contacts		2010 (38.3%)	865 (44.7%)	89 (43.2%)	2964 (40.1%)	
Other/Unknown		315 (6.0%)	116 (6.0%)	31 (15.0%)	462 (6.3%)	
***Ethnicity*, *n(%)***	<.001					
Black		273 (5.2%)	41 (2.1%)	14 (6.8%)	328 (4.4%)	
***AIDS diagnosis*, *n(%)***	<.001					
Yes		600 (11.4%)	249 (12.9%)	60 (29.1%)	909 (12.3%)	
***CCVD diagnosis***, ***n(%)***	<.001					
Yes		375 (7.2%)	223 (11.5%)	56 (27.2%)	654 (8.9%)	
***HBsAg*, *n(%)***	0.890					
Negative		4122 (78.6%)	1536 (79.3%)	163 (79.1%)	5821 (78.8%)	
Positive		230 (4.4%)	81 (4.2%)	11 (5.3%)	322 (4.4%)	
Not tested		891 (17.0%)	319 (16.5%)	32 (15.5%)	1242 (16.8%)	
***HCVAb*, *n(%)***	<.001					
Negative		3020 (57.6%)	1224 (63.2%)	122 (59.2%)	4366 (59.1%)	
Positive		1368 (26.1%)	397 (20.5%)	45 (21.8%)	1810 (24.5%)	
Not tested		855 (16.3%)	315 (16.3%)	39 (18.9%)	1209 (16.4%)	
Hepatitis co-infection*, n(%)	<.001					
No		2780 (53.0%)	1129 (58.3%)	113 (54.9%)	4022 (54.5%)	
Yes		1520 (29.0%)	456 (23.6%)	54 (26.2%)	2030 (27.5%)	
Not tested		943 (18.0%)	351 (18.1%)	39 (18.9%)	1333 (18.1%)	
***Calendar year of baseline***	<.001					
Median (IQR)		2004 (2002, 2009)	2003 (2002, 2009)	2006 (2003, 2010)	2004 (2002, 2009)	
***Age*, *years***	<.001					
Median (IQR)		35 (30, 40)	39 (34, 48)	43 (37, 55)	36 (31, 42)	
***CD4 count*, *cells/mmc***	<.001					
Median (IQR)		449 (288, 646)	453 (270, 660)	361 (124, 551)	447 (280, 647)	
***CD4 count nadir*, *cells/mmc***	<.001					
Median (IQR)		348 (192, 514)	340 (175, 502)	200 (57, 400)	342 (183, 508)	
***CD8 count*, *cells/mmc***	0.454					
Median (IQR)		935 (662, 1288)	944 (654, 1297)	846 (544, 1362)	935 (658, 1291)	
***Viral load*, *log10 copies/mL***	0.014					
Median (IQR)		3.92 (2.43, 4.74)	3.82 (2.30, 4.68)	3.79 (1.70, 4.85)	3.89 (2.35, 4.73)	
***ART-naive*, *n(%)***	<.001					
Yes		998 (24.8%)	422 (26.9%)	48 (28.6%)	1468 (25.5%)	
***Diabetes***, ***n(%)***	<.001					
Yes		84 (1.6%)	56 (2.9%)	16 (7.8%)	156 (2.1%)	
***Smoking***, ***n(%)***	<.001					
No		2071 (39.5%)	846 (43.7%)	104 (50.5%)	3021 (40.9%)	
Yes		2463 (47.0%)	810 (41.8%)	73 (35.4%)	3346 (45.3%)	
Unknown		709 (13.5%)	280 (14.5%)	29 (14.1%)	1018 (13.8%)	
***Total cholesterol***, ***mg/dL***	<.001					
Median (IQR)		167 (141, 196)	179 (150, 210)	179 (145, 215)	170 (143, 201)	
***HDL cholesterol***, ***mg/dL***	0.002					
Median (IQR)		42 (34, 52)	42 (34, 53)	37 (29, 49)	42 (34, 52)	
***Use of statins***, ***n(%)***	0.001					
Yes		75 (1.4%)	41 (2.1%)	9 (4.4%)	125 (1.7%)	
***Use of blood pressure lowering drugs***, ***n(%)***	<.001					
Yes		107 (2.0%)	96 (5.0%)	25 (12.1%)	228 (3.1%)	
***Follow-up***, ***months***	<.001					
Median (IQR)		40 (20, 86)	51 (24, 109)	31 (13, 57)	43 (21, 93)	
***Time from enrolment to first eGFR value*, *months***	0.494					
Median (IQR)		2 (0, 51)	1 (0, 50)	1 (0, 44)	2 (0, 51)	
***egfr (CKD_Epi formula)*, *ml/min/1*.*73m*** ^***2***^	<.001					
Median (IQR)		107.4 (98.87, 114.9)	80.73 (74.43, 85.41)	49.30 (31.35, 55.09)	100.7 (87.21, 111.7)	
***Blood glucose*, *mg/dL***	<.001					
Median (IQR)		86 (80, 94)	88 (81, 96)	90 (80, 100)	87 (80, 95)	

* HCVAb+ or HBsAg+

### Rates of cerebro-cardiovascular and AIDS-related events

Over follow-up, 130 cerebro-cardio-vascular events were observed, of whom 11 were deaths for CCVD (10%), 64 (49.2%) were referable to coronaropathy, 13 (10%) were cerebro-vascular events, 28 (21.5%) were cardiomyopathy and heart failure and 14(10%) classified as other major CCVD events, including coronary artery bypass graft, carotid endarterectomy, or angioplasty.

Patients were followed-up for a total of 35,968 years for an overall incidence of cerebro-cardiovascular events of 3.6 (95% CI: 3.0–4.3) events per 1,000 PYFU. During study follow-up, 311 AIDS-events occurred, of whom 45 (14.5%) were AIDS-related deaths. In this analysis a total follow-up of 35,393 years were accumulated for an overall incidence of AIDS-related events of 8.8 (95% CI:7.8–9.8) events per 1,000 PYFU. A description of the type of cardiovascular and HIV-related events is reported in Table 2.

**Table 2:** Description of event-defining diseases.

Events	Frequency
*Cerebro-cardiovascular events*	*130*
Coronaropathy	64 (49.2%)
Cerebro-vascular events	13 (10%)
Cardiomyopathy and heart failure	28 (21.5%)
Other major CCVD events (including coronary artery bypass graft, carotid endarterectomy, or angioplasty)	14 (10%)
Death from CCVD events	11 (10%)
*AIDS-defining events*	*311*
AIDS-dementia complex	7 (2.7%)
Salmonella bacteremia, recurrent	1(0.3%)
Cervical cancer	15 (5.8%)
Oesophageal candidiasis	33 (12.7%)
Cryptococcosis, extrapulmonary	4 (1.5%)
Cryptosporidiosis	2 (0.7%)
Herpes simplex (bronchitis, pneumonia)	4 (1.5%)
Isosporiasis	1 (0.4%)
Histoplasmosis	2 (0.8%)
Non Hodgkin lymphoma	45 (14.5%)
Disseminated CMV infection	23 (8.8%)
Atypical mycobacteriosis	14 (5.4%)
Progressive multifocal leukoencephalopathy	6 (2.3%)
Recurrent pneumonia	7 (2.7%)
Pneumocystis jiroveci pneumonia	16 (6.1%)
Kaposi’s sarcoma	33 (12.7%)
Cerebral toxoplasmosi	9 (3.5%)
Tuberculosis	31 (11.9%)
Wasting syndrome	14 (5.4%)
Others	7 (2.7%)

### Impaired renal function and risk of cardiovascular events

**Fig 1:**
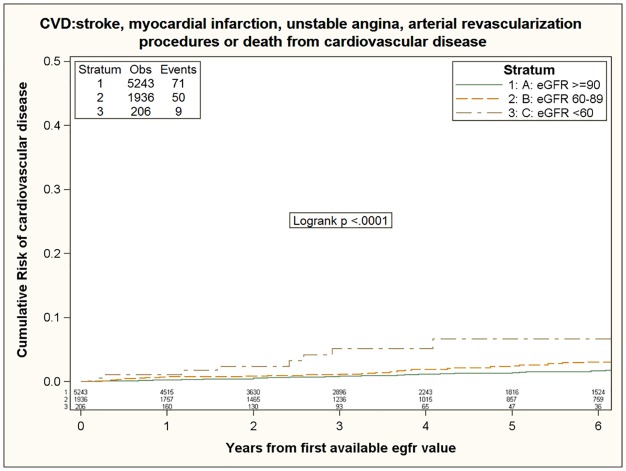
Risk of cerebro-cardiovascular disease during follow-up according to basal eGFR.

According to the three groups with increasing severity of renal dysfunction, we observed a stepwise increase in the rate of cerebro-cardiovascular events, as reported in Table 3. Incidence was 2.91 (95% CI 2.30–3.67), 4.63 (95% CI 3.51–6.11) and 11.9 (95% CI 6.19–22.85) per 1,000 PYFU, among patients with eGFR ≥90, 60–89, <60 ml/min, respectively (Fig 1). In the unadjusted analysis, the incidence rates among patients with reduced renal function (eGFR<60 ml/min) and mild renal impairment (60<eGFR<90 ml/min) were significantly higher than among HIV-infected subjects with normal renal function (eGFR≥90 ml/min), with an unadjusted hazard ratios (HR) of 4.14 (95%CI 2.07–8.29) and 1.58 (95%CI 1.10–2.27). When we evaluated eGFR as a continuous variable we found an unadjusted risk of 5.32 (95%CI 3.02–9.38) per log10 lower eGFR which remained independently associated with higher risk of CCVD in a multivariate model (adjusted HR 3.28; 95%CI 1.47–7.31) including demographic characteristics of patients (age, gender, mode of HIV transmission, nationality and calendar year of baseline), in a second model (adjusted HR 3.71; 95%CI 1.49–9.28) further adjusted for traditional cardio-vascular risk factors (smoking, diabetes, previous AIDS diagnosis, hypertension, lipid lowering drugs, total cholesterol, blood glucose levels, HDL cholesterol), and also in a third model (adjusted HR 7.24; 95%CI 3.05–17.23) further adjusted for HIV-associated characteristics (ART-status, baseline CD4+ and CD8+ T cells count, nadir CD4+ T cell count, baseline HIV-RNA, co-infection with HCV/HBV).

**Table 3:** Rates and hazard ratios of CVD associated with eGFR from fitting a Cox regression model.

					Hazard Ratio (95% CI)				
Baseline eGFR	No. CVD events	PYFU	Rate/1000PYFU	95% CI	Unadjusted	Adjusted(a)	Adjusted(b)	Adjusted(c)	Adjusted(d)
>90	71	24412	2.91	2.30, 3.67	1.00	1.00	1.00	1.00	1.00
60–89	50	10799	4.63	3.51, 6.11	1.58 (1.10, 2.27)	1.18 (0.80, 1.74)	1.72 (1.11, 2.66)	1.63 (1.11, 2.40)	1.56 (0.96, 2.53)
<60	9	756.8	11.9	6.19, 22.85	4.14 (2.07, 8.29)	1.94 (0.93, 4.07)	1.44 (0.44, 4.73)	2.98 (1.28, 6.95)	1.04 (0.30, 3.57)
per log10 lower					5.32 (3.02, 9.38)	3.28 (1.47, 7.31)	3.71 (1.49, 9.28)	7.24 (3.05, 17.23)	3.11 (0.74, 13.13)

^(a)^ Adjusted for age, gender mode of HIV transmission, ethnicity and calendar year of baseline

^(b)^ Adjusted for smoking, diabetes mellitus,previous AIDS diagnosis, blood pressure lowering drugs,lipid lowering drugs, total cholesterol, blood glucose levelsand high density lipoprotein cholesterol

^(c)^ Adjusted for ART-status, baseline CD4, CD8, nadir CD4, baseline HIV-RNA and co-infection with hepatitis B/C

^(d)^ Adjusted for factors in footnotes a-c.

Similarly, mild renal impairment (90<eGFR<60 ml/min) was associated with an increased risk of cerebro-cardiovascular event development (adjusted HR 1.72; 95%CI 1.11–2.66) in the multivariable model adjusted for traditional cardio-vascular risk factors and also after adjustment for HIV associated characteristics (adjusted HR 1.63; 95%CI 1.11–2.40).

Other covariates which resulted independently associated with the risk of CCVD events were: age (HR 1.78 per 10 years older; 95% CI 1.39–2.30) and ethnicity (HR 4.82 black versus Caucasian; 95% CI 1.91–12.16) (Table 4).

**Table 4:** Hazard ratios of CCVD from fitting a Cox regression model (other factors).

	Hazard Ratio of CCVD			
	Unadjusted		Adjusted(e)	
**Gender**: Female vs. male	0.97 (0.66, 1.43)	0.885	1.30 (0.75, 2.26)	0.346
**Mode of HIV Transmission**: Heterosexual contacts		1.00		1.00
IDU	0.92 (0.60, 1.41)	0.711	0.94 (0.44, 2.01)	0.869
Homosexual contacts	0.78 (0.50, 1.21)	0.267	0.92 (0.47, 1.77)	0.797
Other/Unknown	0.90 (0.41, 1.98)	0.796	0.94 (0.33, 2.70)	0.914
***Ethnicity***, Black vs. Caucasian	2.88 (1.51, 5.50)	0.001	4.82 (1.91, 12.16)	<.001
***CCVD diagnosis***,Yes vs. No	3.07 (2.05, 4.60)	<.001	1.35 (0.70, 2.60)	0.367
***HBsAg***, Negative		1.00		1.00
Positive	1.18 (0.80, 1.74)	0.398	1.70 (0.85, 3.37)	0.131
Not tested	1.13 (0.68, 1.88)	0.637	0.39 (0.11, 1.41)	0.152
***HCVAb***, Negative		1.00		1.00
Positive	1.18 (0.80, 1.74)	0.398	1.70 (0.85, 3.37)	0.131
Not tested	1.13 (0.68, 1.88)	0.637	0.39 (0.11, 1.41)	0.152
***Calendar year of baseline*** per more recent	1.03 (0.98, 1.09)	0.246	0.97 (0.88, 1.07)	0.527
***Age***, ***years*** per 10 years older	1.90 (1.63, 2.21)	<.001	1.78 (1.39, 2.30)	<.001
***CD4 count***, ***cells/mmc*** per 100 higher	0.97 (0.91, 1.03)	0.280	1.06 (0.95, 1.19)	0.296
***CD4 count nadir***, ***cells/mmc*** per 100 higher	0.88 (0.81, 0.95)	0.001	0.86 (0.73, 1.01)	0.063
***CD8 count***, ***cells/mmc*** per 100 higher	1.00 (0.99, 1.01)	0.897	0.99 (0.96, 1.03)	0.703
***Viral load***, ***log10 copies/mL*** per log higher	0.94 (0.82, 1.07)	0.349	1.09 (0.89, 1.35)	0.392
***ART-naive***, Yes vs. No	0.66 (0.46, 0.95)	0.024	0.79 (0.43, 1.44)	0.437
***Diabetes***, Yes vs. No	1.81 (0.74, 4.43)	0.192	0.53 (0.15, 1.89)	0.329
***Smoking***, No		1.00		1.00
Yes	1.25 (0.85, 1.82)	0.253	1.41 (0.84, 2.36)	0.193
Unknown	0.80 (0.45, 1.42)	0.442	1.99 (0.73, 5.42)	0.180
***Total cholesterol***, ***mg/dL*** per 100 higher	1.42 (0.99, 2.03)	0.054	1.32 (0.79, 2.19)	0.290
***HDL cholesterol***, ***mg/dL*** per 100 higher	0.17 (0.04, 0.77)	0.022	0.19 (0.03, 1.13)	0.068
***Use of statins***, Yes vs. No	3.95 (2.00, 7.77)	<.001	1.35 (0.50, 3.63)	0.552
***Use of blood pressure lowering drugs***, Yes vs. No	5.37 (3.22, 8.95)	<.001	2.16 (0.96, 4.86)	0.062
***Blood glucose***, ***mg/dL*** per 100 higher	1.28 (0.66, 2.49)	0.470	0.83 (0.29, 2.41)	0.737

### Impaired renal function and risk of AIDS-related events

**Fig 2:**
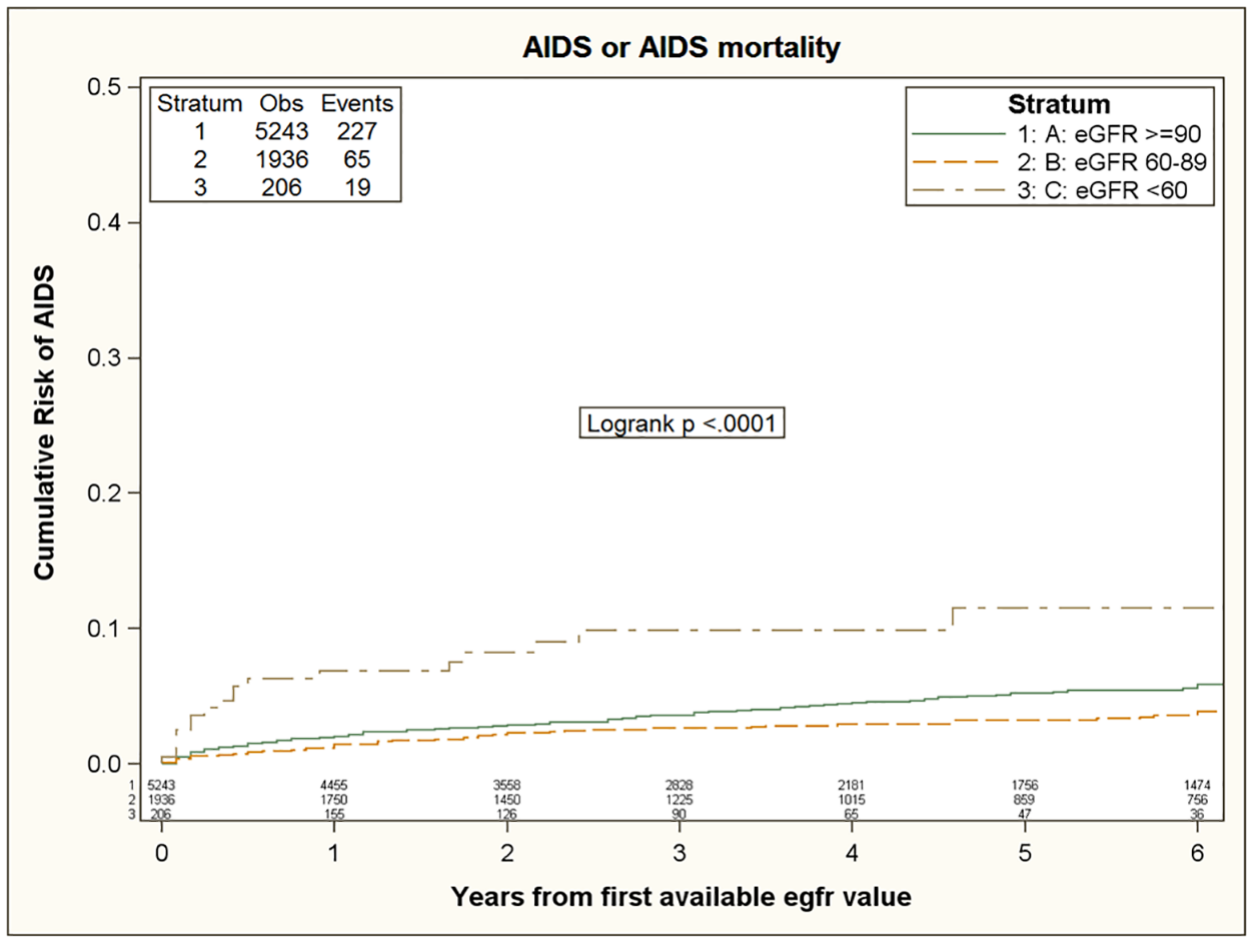
Risk of AIDS-events during follow-up according to basal eGFR.

An increased incidence of AIDS-related events was found in patients with deeply reduced renal function (eGFR<60 ml/min) as reported in Table 5. Incidence was 9.51 (95%CI 8.35–10.83), 6.04 (95%CI 4.74–7.71) and 25.0 (95%CI 15.96–39.22) per 1,000 PYFU, among patients with eGFR ≥90, 60–89, <60 ml/min, respectively (Fig 2). Again, in the unadjusted analysis the incidence rates among patients with reduced renal function (eGFR<60 ml/min) was significantly higher than among HIV-infected subjects with normal renal function (eGFR≥90 ml/min), with an unadjusted hazard ratio of 2.49 (95%CI 1.56–3.97). In contrast, HIV-infected subjects with mild renal impairment (60≤eGFR<90 ml/min) showed decreased incidence rates of HIV-related events as compared to patients with normal renal function (eGFR≥90 ml/min), with an unadjusted hazard ratio of 0.68 (95% CI 0.52–0.90). Reduced renal function (eGFR<60 ml/min) was independently associated with a higher risk of developing HIV-related events (adjusted HR 2.49; 95%CI 1.56–3.97) in a multivariate model adjusted for baseline characteristics (age, gender, mode of HIV transmission, nationality and calendar year of baseline) but did not remain independently associated in a multivariate model adjusted for HIV-associated characteristics (ART-status, baseline CD4+ and CD8+ T cell count, nadir CD4+ T cell count, baseline HIV-RNA, co-infection with HBV/HCV). Interestingly, mild renal impairment was confirmed to be an independent protective factor for HIV-related events (adjusted HR 0.48; 95% CI 0.31–0.75) in the fully adjusted model.

**Table 5:** Rates and hazard ratios of AIDS associated with eGFR from fitting a Cox regression model.

					Hazard Ratio (95% CI)				
Baseline eGFR	No. AIDS events	PYFU	Rate/1000PYFU	95% CI	Unadjusted	Adjusted(a)	Adjusted(b)	Adjusted(c)	Adjusted(d)
>90	227	23880	9.51	8.35, 10.83	1.00	1.00	1.00	1.00	1.00
60–89	65	10754	6.04	4.74, 7.71	0.68 (0.52, 0.90)	0.68 (0.51, 0.90)	0.48 (0.32, 0.72)	0.64 (0.47, 0.88)	0.48 (0.31, 0.75)
<60	19	759.4	25.0	15.96, 39.22	2.49 (1.56, 3.97)	2.20 (1.35, 3.58)	1.71 (0.89, 3.30)	1.90 (1.12, 3.23)	1.26 (0.57, 2.81)

^(a)^ Adjusted for age, gender mode of HIV transmission, ethnicity and calendar year of baseline

^(b)^ Adjusted for smoking, diabetes mellitus,previous AIDS diagnosis, blood pressure lowering drugs,lipid lowering drugs, total cholesterol, blood glucose levelsand high density lipoprotein cholesterol

^(c)^ Adjusted for ART-status, baseline CD4, CD8, nadir CD4, baseline HIV-RNA and co-infection with hepatitis B/C

^(d)^ Adjusted for factors in footnotes a-c.

Other co-variates which resulted independently associated with the risk of cardiovascular events were: female gender (HR 1.64 versus male; 95%CI 1.12–2.42), ethnicity (HR 2.80 Black versus Caucasian; 95%CI 1.49–5.27), previous AIDS diagnosis (HR 1.79; 95%CI 1.19–2.71), baseline HIV-RNA level (HR 1.32 per log10 higher; 95%CI 1.14–1.53) and diabetes diagnosis (HR 3.34 versus no diabetes diagnosis; 95%CI 1.40–7.97) (Table 6).

**Table 6:** Hazard ratios of AIDS from fitting a Cox regression model (other factors).

	Hazard Ratio of AIDS			
	Unadjusted		Adjusted(e)	
**Gender**: Female vs. male	1.16 (0.91, 1.47)	0.242	1.64 (1.12, 2.42)	0.011
**Mode of HIV Transmission**: Heterosexual contacts		1.00		1.00
IDU	1.18 (0.90, 1.55)	0.222	1.66 (0.95, 2.88)	0.073
Homosexual contacts	0.83 (0.63, 1.11)	0.209	1.11 (0.69, 1.79)	0.658
Other/Unknown	0.88 (0.53, 1.49)	0.642	0.58 (0.23, 1.46)	0.249
***Ethnicity***, Black vs. Caucasian	2.25 (1.46, 3.47)	<.001	2.80 (1.49, 5.27)	0.001
***AIDS diagnosis***,Yes vs. No	2.64 (2.05, 3.41)	<.001	1.79 (1.19, 2.71)	0.006
***HBsAg***, Negative		1.00		1.00
Positive	1.45 (0.91, 2.31)	0.119	1.08 (0.54, 2.13)	0.831
Not tested	1.00 (0.74, 1.37)	0.983	0.81 (0.37, 1.76)	0.593
***HCVAb***, Negative		1.00		1.00
Positive	1.16 (0.90, 1.49)	0.257	0.97 (0.57, 1.65)	0.908
Not tested	0.94 (0.67, 1.31)	0.693	1.00 (0.44, 2.28)	0.993
***Calendar year of baseline*** per more recent	1.04 (1.01, 1.07)	0.013	0.99 (0.93, 1.05)	0.651
***Age***, ***years*** per 10 years older	1.09 (0.97, 1.23)	0.131	1.05 (0.86, 1.27)	0.649
***CD4 count***, ***cells/mmc*** per 100 higher	0.78 (0.74, 0.82)	<.001	0.93 (0.84, 1.04)	0.213
***CD4 count nadir***, ***cells/mmc*** per 100 higher	0.80 (0.75, 0.84)	<.001	0.89 (0.77, 1.03)	0.110
***CD8 count***, ***cells/mmc*** per 100 higher	0.99 (0.98, 1.01)	0.495	1.00 (1.00, 1.00)	0.913
***Viral load***, ***log10 copies/mL*** per log higher	1.51 (1.37, 1.67)	<.001	1.32 (1.14, 1.53)	<.001
***ART-naive***, Yes vs. No	1.05 (0.84, 1.31)	0.691	1.09 (0.74, 1.61)	0.664
***Diabetes***, Yes vs. No	1.66 (0.91, 3.03)	0.098	3.34 (1.40, 7.97)	0.007
***Smoking***, No		1.00		1.00
Yes	1.01 (0.80, 1.28)	0.940	1.19 (0.83, 1.70)	0.349
Unknown	0.72 (0.50, 1.05)	0.090	0.93 (0.44, 1.97)	0.854
***Total cholesterol***, ***mg/dL*** per 100 higher	0.43 (0.32, 0.57)	<.001	0.91 (0.61, 1.36)	0.640
***HDL cholesterol***, ***mg/dL*** per 100 higher	0.11 (0.04, 0.31)	<.001	0.37 (0.11, 1.28)	0.117
***Use of statins***, Yes vs. No	0.17 (0.02, 1.18)	0.073	0.27 (0.04, 1.96)	0.196
***Use of blood pressure lowering drugs***, Yes vs. No	0.75 (0.36, 1.60)	0.461	0.52 (0.16, 1.67)	0.272
***Blood glucose***, ***mg/dL*** per 100 higher	1.32 (0.87, 2.01)	0.187	0.64 (0.31, 1.35)	0.246

## Discussion

In a large cohort of antiretroviral treatment-naïve patients seen for care in Italy and starting ART, our primary goal was to evaluate the role of renal function measurement in predicting clinical events, especially cerebro-cardiovascular events, AIDS-related outcomes and even death from both causes.

In the great majority of previously published reports [9–13], kidney disease has been defined by classifying patients in two categories: those with eGFR<60 ml/min and those with eGFR>60 ml/min; in contrast, our study additionally focuses on mild renal impairment, defined as a 60>eGFR<90 ml/min, as before its predictive value has not been extensively investigated despite its high prevalence in HIV- positive populations.

Indeed, while mild renal dysfunction (60<eGFR<90) is a relatively frequent condition in our cohort of HIV-infected patients (26%), we found a low prevalence of severe renal impairment (3% of population had eGFR<60ml/min) at baseline. This was anticipated as people are a relatively healthy population enrolled when they are antiretroviral-naïve by study design.

Our study highlights the existence of a strong link between kidney function and CCVD risk in HIV-infected individuals. Indeed, we observed a marked gradient in the association between eGFR levels and the risk of cerebro-cardiovascular disease, with an intermediate excess of risk of CCVD in patients with mild renal impairment (eGFR 60–89 ml/min) and a greater risk in patients with severe chronic disease (eGFR< 60 ml/min). Importantly, we found that lower eGFR was associated with a significantly increased risk of CCVD even after adjustment for baseline demographic characteristics of patients, HIV-associated factors and traditional cardiovascular risk factors (smoking, diabetes, hypertension, use of lipid lowering drugs, total and HDL cholesterol values, blood glucose levels). This held true both in the analysis using the categorical variable comparing different levels of renal impairment and in the analysis using eGFR as continuous in the log scale.

Thus, our results confirm that reduced impaired renal function is an important risk factor for cerebro-cardiovascular events in the HIV-population. As outlined above, one of the key finding is that, compared to other studies investigating the association between kidney function and the risk of CCVD^9^, this is the first to identify HIV-infected patients with mild renal impairment (90<eGFR<60) as a population at increased risk of cerebro-cardiovascular morbidity and mortality.

This result is however consistent with that of multiple studies conducted in general population, which showed that kidney disease is associated with dyslipidemia, anemia, left ventricular hypertrophy, arterial stiffness, inflammation and endothelial dysfunction [17].

Conversely, in our analysis, HIV-associated parameters do not seem to play a substantial role in the development of CCVD events; indeed in our population only age and ethnicity were independent markers of CCVD risk, while poor immunological condition, represented by CD4 cells count and CD4 cells nadir, did not predict cerebro-cardiovascular events. These finding differ from other studies which found that lower CD4+ count, together with traditional CCVD risk factors and duration of ART are associated with increased incidence of CCVD events [18–19]. But recent data from D.A.D. And COHERE show the same thing i.e. That CD4 count does not predict risk of MI and CVD events in general. This difference might be explained by the characteristics of the patients in our cohort (CD4 count were high on average at the date in which the first eGFR was measured) and the analysis takes into account only baseline CD4 count and not the current value.

Our study highlights the importance of estimation of filtrate through creatinine measurement as a clinical tool for identifying HIV-infected individuals at elevated risk for cerebro-cardiovascular events, and strengthens the fact that managing traditional risk factors in HIV population with renal impairment should be a priority.

Kidney disease is also feared as a predisposing condition for progression toward AIDS and death. In our analysis, initial serious renal impairment (eGFR<60) seems to be associated with an increase in number of AIDS related illnesses. However, this link was not confirmed after adjustment for the presence or history of antiretroviral treatment, CD4+ T cells nadir, baseline CD4+ and CD8+ T cells count, and baseline HIV viral load together with chronic hepatitis co-infections. This is consistent with the results of *Sczech et al* who found that dipstick proteinuria but not inverse creatinine was significantly associated with the development of AIDS-defining illness [10]. Unfortunately dipstick proteinuria was not available for our patients. This finding is suggestive for the many sided clinical picture of patients who usually develops AIDS defining illnesses in which HIV-related parameters, as a mirror of immunological condition, play a pivotal role in determining collapse toward AIDS development. On the other hand, advanced HIV disease is also associated with renal dysfunction and it is therefore a confounder for the association between renal disease and risk of AIDS and AIDS-related death. All the parameters in our analysis have been measured concomitantly at baseline and therefore it is difficult to understand dynamic mechanisms that might have led to disease progression. Even in a situation in which prospective measures of both creatinine and CD4 count are considered is often difficult to establish links of causality. Nevertheless, it is conceivable that renal impairment is likely to be a consequence and not the cause of a worse immunological situation. Of interest, participants with mild dysfunction were at lower risk of HIV progression and the association remained significant after controlling for a number of potential confounding factors. This might be due to the combined effect of low impact of renal disease on long term HIV disease progression and the fact that these patients were more able to tolerate and respond well to antiretroviral treatment. Some of this question might be answered by including time-dependent confounding but more sophisticated methods than standard survival analysis are needed to disentangle the complex confounding issue of time-dependent markers affected by prior use of ART [20]

Our results, however, differ from those of other studies assessing and justifying the development of renal abnormalities as predictors for AIDS progression [10–11]. *Gupta et al*, found that lower renal function defined as eGFR < 60 ml/min was independently associated with an increased risk of HIV disease progression [12]. Reasons for the discrepancy are unclear but might be attributable to the different endpoint used (time to developing either a CD4 count <200 cells/mm3 or WHO stage 3 or 4 disease) clinical setting, patients ethnicity or a combination of these.

In a recent analysis Lucas G et al evaluated the association of eGFR, estimated by three different equations (Cockcroft/Gault, MDRD and CKD-Epi with and withous the inclusion of cystatin C besides creatinine), with clinical events in HIV-infected patients in the SMART study and found that eGFR estimates using plasma cystatin C had stronger associations with mortality, cardiovascular events and opportunistic diseases than eGFR based on plasma creatinine [21]. Indeed, one limit of our study is the unavailability of other markers of kidney damage, expecially in patients with eGFR above 60 ml/min, for whom CKD-EPI, like other existing GFR-estimating equations have increasing bias. Unfortunately, plasma cystatin C was not available in our patients and its predictive value in patients receiving ART should be further investigated.

In conclusion, in this large cohort of HIV-infected patients seen for care in Italy, we observed a strong association between levels of eGFR and cerebro-cardiovascular diseases, while severe renal impairment did not predict HIV-related events. A major strength of our study is the analysis of the predictive role of mild renal impairment. It is important to increase our understanding of the mechanisms leading to severe non-AIDS morbidity anf mortality in the context of the emerging clinical challenge of treating an ageing HIV-infected population. These results may improve clinical management of HIV-infected patients by helping clinicians in identifyng people at higher risk of CCVD and provide a rationale for the evaluation of therapeutic strategies aimed to ameliorate patients' CCVD risk profile.
